# Impact of a Public Health Emergency on Behavior, Stress, Anxiety and Glycemic Control in Patients With Pancreas or Islet Transplantation for Type 1 Diabetes

**DOI:** 10.3389/ti.2024.12278

**Published:** 2024-03-27

**Authors:** Cyril P. Landstra, Merel M. Ruissen, Hannah Regeer, Michiel F. Nijhoff, Bart E. P. B. Ballieux, Paul J. M. van der Boog, Aiko P. J. de Vries, Sasja D. Huisman, Eelco J. P. de Koning

**Affiliations:** ^1^ Department of Internal Medicine, Leiden University Medical Center, Leiden, Netherlands; ^2^ Department of Biomedical Data Sciences, Section Medical Decision Making, Leiden University Medical Center, Leiden, Netherlands; ^3^ Transplantation Center, Leiden University Medical Center, Leiden, Netherlands; ^4^ Department of Clinical Chemistry and Laboratory Medicine, Leiden University Medical Center, Leiden, Netherlands

**Keywords:** COVID-19, type 1 diabetes, islet transplantation, pancreas transplantation, behavior, stress, anxiety, glycemic control

## Abstract

A public health emergency such as the COVID-19 pandemic has behavioral, mental and physical implications in patients with type 1 diabetes (T1D). To what extent the presence of a transplant further increases this burden is not known. Therefore, we compared T1D patients with an islet or pancreas transplant (β-cell Tx; *n* = 51) to control T1D patients (*n* = 272). Fear of coronavirus infection was higher in those with β-cell Tx than without (Visual Analogue Scale 5.0 (3.0–7.0) vs. 3.0 (2.0–5.0), *p* = 0.004) and social isolation behavior was more stringent (45.8% vs. 14.0% reported not leaving the house, *p* < 0.001). A previous β-cell Tx was the most important predictor of at-home isolation. Glycemic control worsened in patients with β-cell Tx, but improved in control patients (ΔHbA1c +1.67 ± 8.74 vs. −1.72 ± 6.15 mmol/mol, *p* = 0.006; ΔTime-In-Range during continuous glucose monitoring −4.5% (−6.0%–1.5%) vs. +3.0% (−2.0%–6.0%), *p* = 0.038). Fewer patients with β-cell Tx reported easier glycemic control during lockdown (10.4% vs. 22.6%, *p* = 0.015). All T1D patients, regardless of transplantation status, experienced stress (33.4%), anxiety (27.9%), decreased physical activity (42.0%), weight gain (40.5%), and increased insulin requirements (29.7%). In conclusion, T1D patients with β-cell Tx are increasingly affected by a viral pandemic lockdown with higher fear of infection, more stringent social isolation behavior and deterioration of glycemic control.

This trial has been registered in the clinicaltrials.gov registry under identifying number NCT05977205 (URL: https://clinicaltrials.gov/study/NCT05977205).

## Graphical Abstract

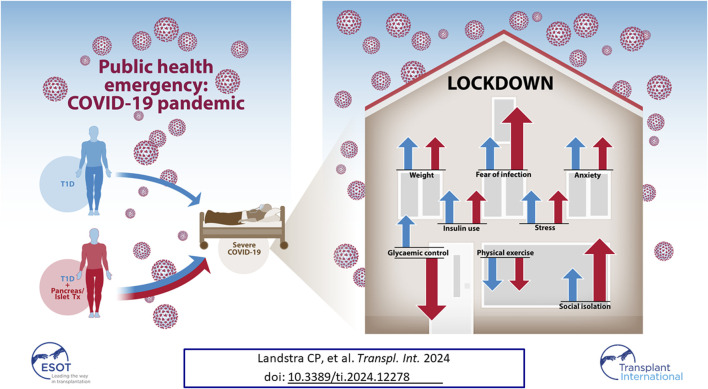

## Introduction

As the most recent public health emergency of international concern, as declared by the World Health Organization, the Severe Acute Respiratory Syndrome Coronavirus 2 (SARS-CoV-2) pandemic has resulted in a rapid increase in morbidity and mortality. Up until now, almost 775 million cases have been reported worldwide, resulting in more than 7 million deaths [1]. In an attempt to control the outbreak, governments of many countries implemented different quarantine strategies, varying from a complete lockdown and curfews to social distancing measures and a ban on public and social events. The predominant incentives for these measures were to relieve the extraordinary pressure put on already strained healthcare systems and to protect vulnerable patient groups from contracting COVID-19 [2, 3]. These restrictive measures required major adaptation in behavior and resulted in considerable disruptions in daily life, known to influence both mental and physical health [4, 5].

Some patients are at particularly high risk of a more severe course of the disease and mortality. These include patients with older age, obesity, hypertension, cardiovascular disease (CVD), chronic kidney disease, a history of organ transplantation and diabetes mellitus [6–8]. These risk groups, including patients with type 1 diabetes mellitus (T1D), were warned to be particularly stringent in adhering to quarantine measures [9, 10]. Therefore, an increased adverse effect on mental and physical health may be expected in these patients. Moreover, emotional distress, anxiety and change in daily structures are known to influence glycemic control [11–13]. Indeed, we recently reported a significant impact on psychological outcomes including stress and anxiety in patients with T1D [14]. Unexpectedly, however, glycemic control showed improvement in patients with T1D in different countries during the COVID-19 lockdown, underpinning the multitude of factors that influence glycemic control [14–18].

A specific subgroup of patients with T1D are those with severely complicated diabetes who have received a pancreas or islet transplantation (β-cell Tx) [19, 20]. In addition to the higher risk of severe COVID-19 in T1D alone, these patients use immunosuppression, providing another key risk factor for a severe course of COVID-19 [7, 21–23]. Therefore, we not only expected an even greater impact of COVID-19 and the subsequent lockdown on mental and physical health in these patients, but also more stringent social isolation behavior which could adversely affect glycemic control [24]. As there is a lack of data on these aspects in patients with a previous pancreas or islet transplantation, we studied the impact of a COVID-19 nationwide partial lockdown in this patient group.

## Patients and Methods

### Design and Patients

For this single-center observational cross-sectional study, patients with T1D in care at the Leiden University Medical Center (LUMC) were asked to participate in the study. From the LUMC Transplant Center, patients with T1D who had received an islet or pancreas [including simultaneous pancreas-kidney (SPK)] transplantation were included. This group is referred to as the “β-cell Tx” group in this manuscript. In the Netherlands, the government implemented the first partial lockdown on March 15th, 2020, which is considered the start of the lockdown period in this study. Social distancing measures were implemented, gatherings were banned and people were strongly advised to stay at home, with the exception of individuals working in vital areas of society. Public spaces, non-essential shops, restaurants, bars, and schools were closed [25].

The patients with T1D without β-cell transplantation were part of a larger study into the effects of the COVID-19 lockdown in patients with type 1 and type 2 diabetes mellitus [14]. All islet transplant recipients as well as pancreas transplant recipients with less than optimal transplant function according to Igls criteria were eligible, in order to determine the effect of the lockdown period on glycemic control [26]. Information on the Igls criteria and how they score graft function can be found in our Supplementary Material (Supplementary Table S1). Further inclusion criteria included adult age (≥18 years), sufficient understanding of the Dutch language, the ability to perform a fingerstick HbA1c measurement and the ability to complete an online questionnaire [14]. Exclusion criteria included pregnancy, recent diagnosis with any malignancy (≤6 months), current immuno- or chemotherapy and admission at a hospital or rehabilitation center. Additionally, for this analysis, patients with T1D without a previous β-cell Tx that used steroids and/or other immunosuppressive agents at the time of inclusion were excluded from the analyses, as well as patients with a previous β-cell Tx that did not use steroids and/or other immunosuppressive at the time of inclusion. Reasons for (not) using immunosuppressive agents and more information on the exclusion of these patients can be found in the flowchart in Supplementary Figure S1. Since a recent start of flash glucose monitoring (FGM) or CGM can improve glycemic control, all patients (i.e., both T1D with as well as without β-cell Tx) that started using FGM or CGM within 2 months of the start of the lockdown were excluded from flash or continuous glucose sensor data analysis as well as HbA1c analysis.

Prior to the start, this study was approved by the Medical Ethical Committee of Leiden—Den Haag—Delft under the Medical Research Involving Human Subjects Act, under reference number NL73778.058.20. The study was registered in the clinicaltrials.gov registry under identification number NCT05977205. Written informed consent was provided by all participants.

### Data Collection

Digital questionnaires were sent out and data was collected using Castor (Castor Electronic Data Capture, Ciwit BV, Amsterdam, the Netherlands). For HbA1c measurements, a validated capillary blood sampling set containing a small tube, lancet and medical return envelope was sent to each participant to prevent unnecessary visits to the hospital [27]. Patients were instructed to fill the tube with a few drops of blood and return it by mail to the LUMC, where HbA1c was analyzed on the day of arrival using a Tosoh G8 HbA1c analyzer. Other patient data (including other laboratory values and patient medical history) were extracted from electronic patient records. These data were all collected during the lockdown period, 8–11 weeks after the start of the lockdown.

### Outcome Measures

The primary outcome measure was the difference in HbA1c that was measured before and during the lockdown. Several secondary outcome measures were defined. For patients with FGM or CGM, glucose monitoring data were assessed for two different two-week time periods, before and at the end of the lockdown (February 24th—March 8th, 2020 and April 23rd—May 7th, 2020, respectively). For these two-week time frames, time in range (TIR; % of time between 3.9–10.0 mmol/L), time above range (TAR; % of time ≥10.0 mmol/L), time below range (TBR; % of time <3.9 mmol/L), the coefficient of variation (CV), time of active use (% of time) and for patients with FGM also the average amount of scans per day (n) were evaluated. Level of education was categorized as low (primary education), middle (practical training and education; lower and senior preparatory vocational education; senior general secondary education), and high [higher professional education; (pre-) university and (post-) doctoral studies]. Psychological distress was assessed by the Perceived Stress Scale (PSS), in which a score of ≥14 indicates moderate stress [28]. The online questionnaire also included items on daily routines, physical activity and reported (changes in) glycemic control, medication use and stress and anxiety regarding COVID-19 (Supplementary Material Sl). For patients with β-cell Tx, graft function was assessed using the Igls criteria to be optimal, good, marginal or failed. Treatment success was determined as optimal or good graft function, while treatment failure was determined as marginal or failed graft function [26]. As is the case in any similar study, selection bias may have played a role as the approached potential participants could voluntarily decide to participate or not. Recall bias may have been present, but is minimized due to the relatively short interval between the start of the lockdown and the questionnaires.

### Data Analysis

Statistical analyses were performed using IBM^®^ SPSS^©^ Statistics version 25 (IBM Corporation, Armonk, New York, United States of America). Normality of distribution was assessed by the Kolmogorov-Smirnov test of normality as well as through visual histogram distribution evaluation. An unpaired *t*-test was used for comparing normally distributed numerical variables in patients with T1D with and without β-cell Tx. A paired *t*-test was used for comparing normally distributed numerical variables in patients before and during the lockdown period. Mann-Whitney U test was used for comparing non-parametric numerical variables in patients with versus without β-cell Tx and Wilcoxon Signed Rank test for non-parametric numerical variables in patients before and during the lockdown period. For categorical variables, χ^2^ test was used for comparing unpaired and Wilcoxon Signed Rank test for paired variables. One-way ANOVA was used for comparing normally distributed numerical variables, and Kruskall-Wallis for comparing non-parametric numerical variables in patients with islet transplantation (ITx), solitary pancreas transplantation (PTx) and SPK. Univariable linear regressions were used to assess associations for univariable numerical outcomes of interest (including potential confounders), all variables that reached statistical significance in the univariable linear regression analysis were added to the multivariable linear regression model. Univariable logistic regressions were used for univariable categorical outcomes, all variables that reached statistical significance in the univariable logistic regression analysis were added to the multivariable logistic regression model. Missing data were considered to be missing at random, cases with missing data were excluded from the particular analysis where data was missing and were not excluded from all analyses. Normally distributed numerical variables are expressed as mean ± standard deviation (SD), non-parametric numerical variables as median [first quartile (Q1)—third quartile (Q3)]. Calculated differences (Δ) are expressed as mean difference ±standard error of the difference. Categorical variables are expressed as number of cases (percentage of patient population). A *p*-value of <0.05 was considered statistically significant.

## Results

### Patients and Characteristics

A total of 51 patients with T1D with a previous β-cell Tx and 272 patients with T1D without β-cell Tx were eligible for this study and provided signed written informed consent. Of the 51 β-cell Tx recipients, 19 (37.3%) received islets, 5 (9.8%) solitary pancreas, and 27 (52.9%) SPK transplants. Baseline characteristics of patients with T1D with and without β-cell Tx are described in Table 1. Patients with β-cell Tx had a lower BMI, longer diabetes duration with more diabetes-related complications, and higher blood pressure with more anti-hypertensive medication. Other important risk factors for a severe course of COVID-19, such as age, sex, smoking and pulmonary comorbidities, were not different between the groups. A total of 33/50 (66.0%) of patients with β-cell Tx receive triple immunosuppression, mostly consisting of tacrolimus, mycophenolate mofetil and prednisolone (5 mg/d). Data from FGM/CGM were available in a total of 99/323 (30.7%) patients (12/51 (23.5%) of patients with β-cell Tx and 87/272 (32.0%) of patients with T1D). A total of 305/323 (94.4%) patients (48/51 (94.1%) of patients with β-cell Tx and 257/272 (94.5%) of patients with T1D) completed the online questionnaire on daily routines, physical activity, stress and anxiety, glycemic control and medication.

**TABLE 1:** Baseline characteristics.

	β-cell Tx	T1D	*p*-value
n	51	272	NA
Age, years (median, Q1–Q3)	55 (48–59)	53 (37–62)	0.103
Sex, female (n, %)	20/51 (39.2%)	126/272 (46.3%)	0.349
BMI, kg/m^2^ (median, Q1–Q3)	23.3 (20.9–27.4)	25.2 (23.0–28.0)	**0.016**
Level of education (n, %)^a^			
* Low*	3/47 (6.4%)	9/256 (3.5%)	**0.002**
* Middle*	29/47 (61.7%)	95/256 (37.1%)	
* High*	15/47 (31.9%)	152/256 (59.4%)	
Living situation (n, %)			
* Alone*	8/48 (16.7%)	37/257 (14.4%)	0.988
* Co-habitant*	40/48 (83.3%)	220/257 (85.6%)	
Duration of diabetes, years (median, Q1—Q3)	42 (34–48)	38 (15–39)	**<0.001**
Antihyperglycemic therapy (n, %)			
* None*	0/51 (0.0%)	0/255 (0.0%)	**<0.001**
* Oral antihyperglycemic agents only*	6/51 (11.8%)	0/255 (0.0%)	
* Insulin: long-acting only*	9/51 (17.6%)	9/255 (3.5%)	
* Insulin: basal-bolus therapy*	36/51 (70.6%)	246/255 (96.5%)	
Glucose monitoring (n, %)			
* None*	6/48 (12.5%)	3/257 (1.2%)	**<0.001**
* Blood glucose monitoring only*	19/48 (39.6%)	61/257 (23.7%)	
* Flash or continuous glucose monitoring*	23/48 (47.9%)	193/257 (75.1%)	
Complications (n, %)			
* Retinopathy*	44/51 (86.3%)	182/269 (67.7%)	**0.007**
* Lasercoagulation*	40/50 (80.0%)	57/268 (21.3%)	**<0.001**
* eGFR ≥ G2* ^b^	49/50 (98.0%)	116/262 (44.3%)	**<0.001**
* Albuminuria (A2-A3)*	29/41 (70.7%)	24/49 (49.0%)	0.090
* Peripheral neuropathy*	35/50 (70.0%)	64/264 (24.2%)	**<0.001**
* Cardiovascular complications* ^c^	35/51 (68.6%)	44/272 (16.2%)	**<0.001**
Immunosuppressive regimen (n, %)			
* None*	0/51 (0.0%)	NA	NA
* Tac + MMF*	8/50 (16.0%)		
* Tac + pred*	5/50 (10.0%)		
* Tac + MMF + pred*	28/50 (56.0%)		
* Tac + pred + other*	5/50 (10.0%)		
* Other*	4/50 (8.0%)		
Type of β-cell transplantation			
* Islets*	19/51 (37.3%)	NA	NA
* Solitary pancreas*	5/51 (9.8%)		
* SPK*	27/51 (52.9%)		
Igls score (n, %)			
* Failure*	13/51 (25.5%)	NA	NA
* Marginal*	16/51 (31.4%)		
* Good*	21/51 (41.2%)		
* Optimal*	1/51 (1.9%)		
Blood pressure, mmHg (median, Q1—Q3)			
* Systolic blood pressure*	146 (134–160)	130 (121–140)	**<0.001**
* Diastolic blood pressure*	80 (77–83)	78 (73–82)	0.085
Anti-hypertensive medication (n, %)^d^	37/51 (72.5%)	102/272 (37.5%)	**<0.001**
LDL cholesterol, mmol/mol (median, Q1–Q3)	2.22 (1.91–2.51)	2.30 (1.84–2.89)	0.306
Lipid lowering medication (n, %)			
* Statins*	30/51 (58.8%)	105/269 (39.0%)	**0.009**
* Ezetimibe*	2/51 (3.9%)	10/269 (3.7%)	0.944
Smoking (n, %)^e^			
* No*	47/51 (92.2%)	231/259 (89.2%)	0.491
* Occasional*	0/51 (0.0%)	7/259 (2.7%)	
* Regular*	4/51 (7.8%)	21/259 (8.1%)	
Pulmonary comorbidities (n, %)^f^	2/51 (3.9%)	16/270 (5.9%)	0.568

β-cell Tx, β-cell transplantation; T1D, type 1 diabetes; BMI, body mass index; eGFR, estimated glomerular filtration rate; Tac, tacrolimus; MMF, mycophenolate mofetil; pred, prednisolone; SPK, simultaneous pancreas-kidney transplantation; LDL, low-density lipoprotein.

Bold *p*-values are considered statistically significant (*p* < 0.05).

^a^ Level of education: low (primary education); middle (practical training and education; lower and senior preparatory vocational education; senior general secondary education); and high [higher professional education; (pre-) university and (post-) doctoral studies].

^b^ Chronic Kidney Disease Guideline classification: eGFR ≥G2 = eGFR ≤ 89 mL/min/1.73 m^2^.

^c^ History of myocardial infarction, percutaneous coronary intervention, peripheral vascular disease, stroke, transient ischemic attack, heart failure, amputation of limbs (toe/foot/leg).

^d^ Use of any or more of the following anti-hypertensive medication: angiotensin converting enzyme (ACE) inhibitor, angiotensin receptor blocker (ARB), calcium antagonist, alpha blocker, beta blocker, thiazide diuretics, spironolactone.

^e^ Occasional smoking ≥1x/week; regular smoking ≥1x/day.

^f^ History of pulmonary comorbidities: asthma, chronic obstructive pulmonary disease (COPD) or fibrosis.

### COVID-19 Fear and Social Isolation Behavior

**FIGURE 1:**
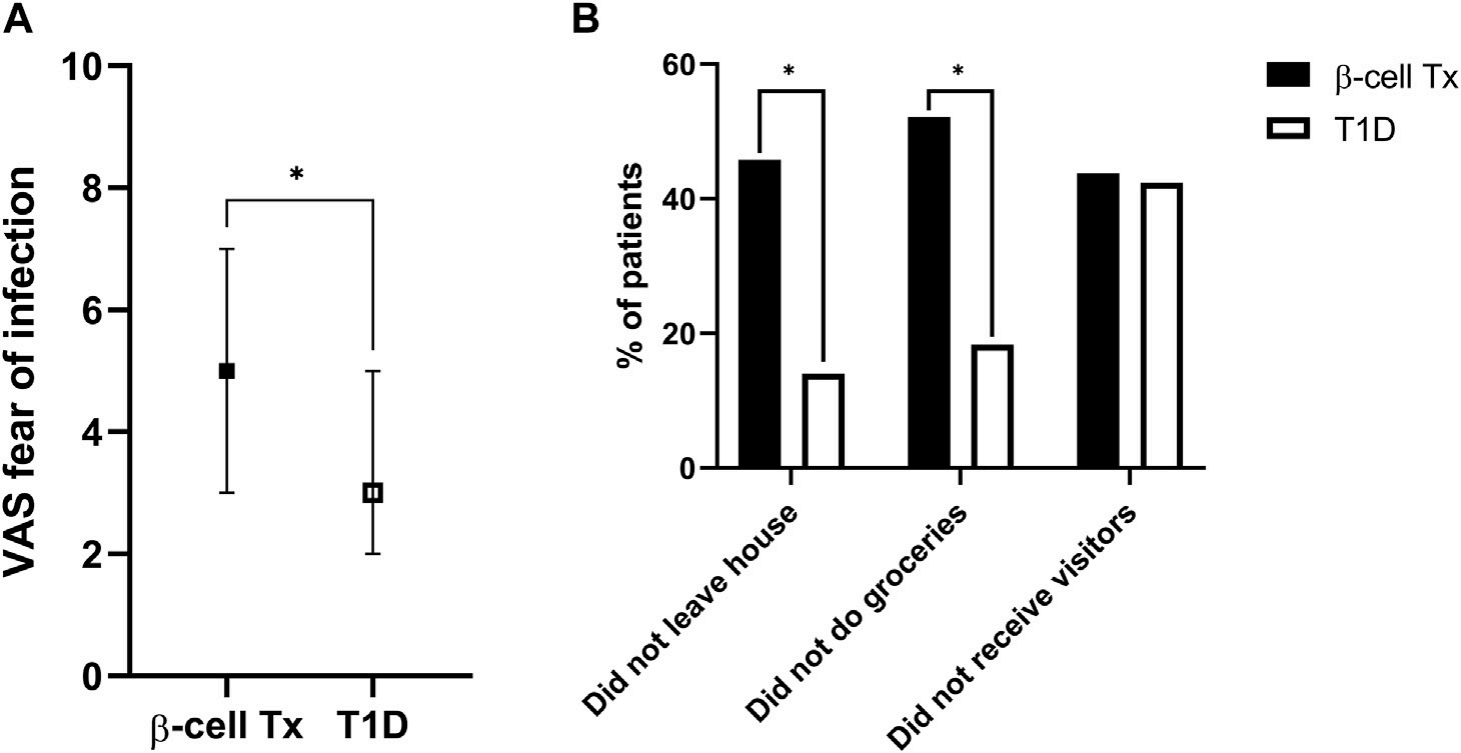
Fear of infection and COVID-19 social isolation behavior **(A)**. Fear of contracting COVID-19 (visual analogue scale, ranging from 1–10) **(B)**. Social isolation behavior during the COVID-19 lockdown: percentage of patients not leaving the house, not doing their own groceries and not allowing visitors inside their homes VAS, visual analogue scale; β-cell Tx, β-cell transplantation; T1D, type 1 diabetes *p < 0.05.

Patients with β-cell Tx had significantly higher fear of COVID-19 infection as compared to patients with T1D alone (Figure 1A; VAS 5.0 (3.0–7.0) vs. 3.0 (2.0–5.0), *p* = 0.004). They also behaved differently with regard to social isolation and adherence to the lockdown measures, with 52.1% vs. 18.3% (*p* < 0.001) reporting not going out for groceries and 45.8% vs. 14.0% (*p* < 0.001) reporting not leaving the house at all (Figure 1B). Univariate analysis demonstrated that a β-cell transplantation, a history of CVD and higher fear of infection with COVID-19 were univariable significant predictors of not leaving the house (Table 2). The only significant independent predictor that remained after multivariable regression analysis was a previous β-cell transplantation, with an odds ratio (OR) of 4.275 (95% CI 1.919–9.537, *p* < 0.001). Age, sex, BMI, level of education, pre-lockdown HbA1c, blood pressure, and pulmonary comorbidities were not found to be associated with isolating at home.

**TABLE 2:** Univariable and multivariable predictors of not leaving the house in patients with type 1 diabetes with and without β-cell transplantation.

	Univariable logistic regression			Multivariable logistic regression		
	B	R^2^ (95% CI)	*p*-value	B	R^2^ (95% CI)	*p*-value
Age, years	0.009	1.009 (0.989–1.030)	0.384			
Sex (ref. female)	−0.239	0.787 (0.444–1.395)	0.412			
BMI, kg/m^2^	−0.078	0.925 (0.852–1.004)	0.061			
Level of education (ref. middle)						
*Low*	−0.182	0.833 (0.171–4.055)	0.821			
*High*	−0.012	0.988 (0.548–1.780)	0.967			
HbA1c pre-L, mmol/mol Hb	0.004	1.004 (0.981–1.026)	0.758			
β-cell Tx (ref. T1D)	1.648	5.194 (2.663–10.133)	**<0.001**	1.453	4.275 (1.919–9.537)	**<0.001**
Pulmonary comorbidities	−0.529	0.589 (0.130–2.668)	0.492			
Cardiovascular disease	0.879	2.408 (1.302–4.451)	**0.005**	0.183	1.201 (0.559–2.580)	0.639
Systolic blood pressure	0.015	1.015 (1.000–1.030)	0.055			
VAS fear of infection	0.137	1.147 (1.019–1.291)	**0.023**	0.092	1.096 (0.968–1.241)	0.148

Ref, reference; BMI, body mass index; Hb, hemoglobin; pre-L, pre-lockdown; β-cell Tx, β-cell transplantation; T1D, type 1 diabetes mellitus; VAS, visual analogue scale.

Bold *p*-values are considered statistically significant (*p* < 0.05).

### Impact on Glycemic Control

**FIGURE 2:**
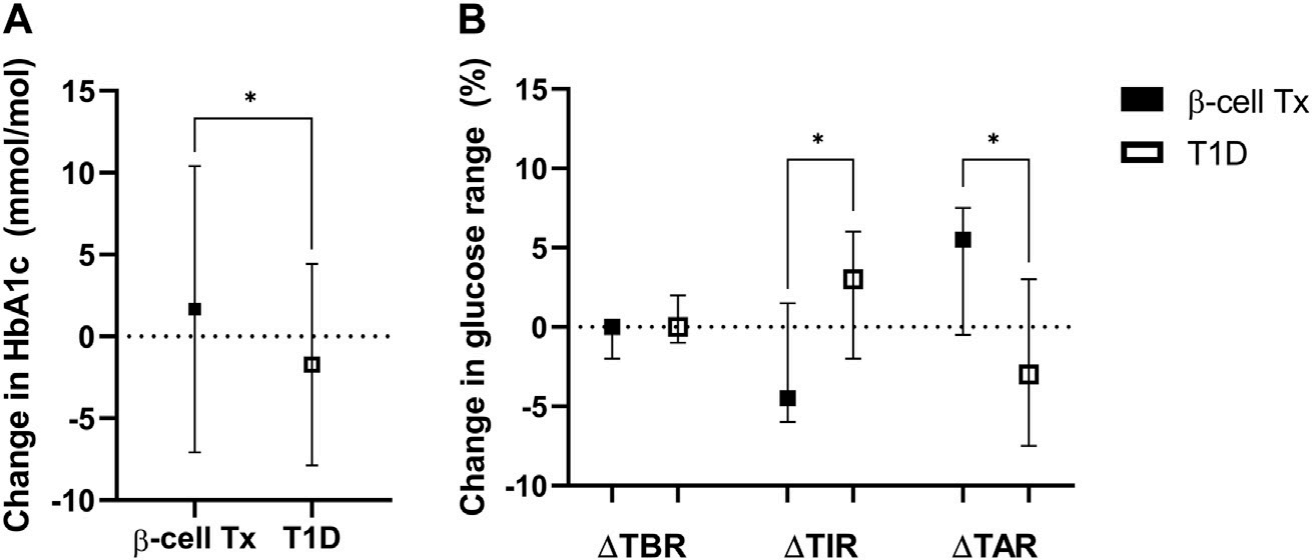
Change in glycemic control over the COVID-19 lockdown period **(A)**. Change in HbA1c (mmol/mol Hb) **(B)**. Change in continuous glucose monitoring metrics of glucose regulation: percentage of time below range (TBR; % of time <3.9 mmol/L), time in range (TIR; % of time between 3.9–10 mmol/L) and time above range (TAR; % of time ≥10.0 mmol/L) β-cell Tx, β-cell transplantation; T1D, type 1 diabetes *p < 0.05.

**FIGURE 3:**
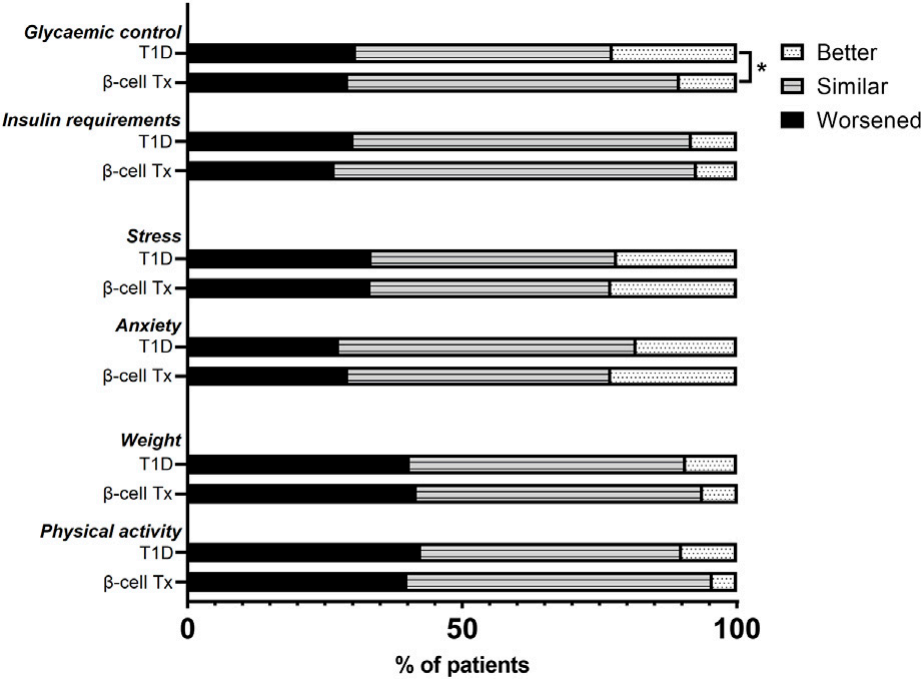
Changes in glycemic control, insulin requirements, stress, anxiety, weight, and physical activity over the COVID-19 lockdown period in patients with type 1 diabetes with and without β-cell transplantation. β-cell Tx, β-cell transplantation; T1D, type 1 diabetes *p < 0.05.

HbA1c during lockdown (measured at a median of 9.3 (8.7–10.3) weeks after the initiation of the lockdown) was compared to the last known pre-lockdown HbA1c (measured at a median of 9.1 (4.4–21.0) weeks before the lockdown). In patients with β-cell Tx, HbA1c increased by 1.7 ± 8.7 mmol/mol Hb (54.3 mmol/mol Hb pre-lockdown to 56.0 mmol/mol Hb during the lockdown). In patients with T1D HbA1c decreased by −1.7 ± 6.1 mmol/mol Hb (60.5 mmol/mol Hb pre-lockdown to 58.8 mmol/mol Hb during the lockdown) (Figure 2A; *p* = 0.006). These findings were reflected in glucose monitoring data showing a reduction in time in range (TIR) and an increase in time above range (TAR) in patients with β-cell Tx, while patients with T1D showed an increase in TIR and reduction in TAR (Figure 2B; ΔTIR β-cell Tx −4.5% (−6.0% – 1.5%) vs. T1D 3.0% (−2.0% – 6.0%), *p* = 0.038; ΔTAR β-cell Tx 5.5% (−0.5% – 7.5%) vs. T1D −3.0% (−7.5% – 3.0%), *p* = 0.025). There was no significant difference in ΔHbA1c or CGM outcomes between patients with different types of β-cell transplantation (i.e., ITx, PTx or SPK; Supplementary Table S2). In patients with T1D with and without β-cell Tx, 26.8% vs. 30.2% (*p* = 0.871) reported administration of more insulin compared to before the COVID-19 lockdown (Figure 3; Supplementary Table S3). In terms of reported glucose regulation, a similar number of patients (29.2% vs. 30.7%) reported more difficulty with glycemic control, but in patients with β-cell Tx compared to T1D alone, less patients reported that they found it easier to regulate their blood glucose levels over the lockdown period (10.4% vs. 22.6%; *p* = 0.015; Figure 3; Supplementary Table S3). Within the group of patients with β-cell Tx, these outcomes did not differ between different β-cell replacement modalities (Supplementary Table S4). Univariate analysis showed that pre-lockdown HbA1c (OR 0.918; 95% CI 0.858–0.983; *p* = 0.014) and treatment success, as determined by the Igls score (OR 5.571; 95% CI 1.297–23.934; *p* = 0.021), were significant predictors for a deterioration in HbA1c for patients with β-cell Tx. However, in a multivariable model, both variables lost statistical significance (Supplementary Table S5).

### Impact on Stress, Anxiety, Weight and Physical Exercise

In patients with T1D with and without β-cell Tx, increased anxiety (29.2% vs. 27.6%, *p* = 0.677) and stress (33.3% vs. 33.5%, *p* = 0.984) during the COVID-19 lockdown as compared to before the lockdown was reported in similarly high numbers (Figure 3; Supplementary Table S3), with a PSS of 14.04 ± 7.05 vs. 13.69 ± 6.16 (*p* = 0.722), indicating moderate perceived stress in patients with β-cell Tx and low perceived stress in patients with T1D alone. Furthermore, 41.7% vs. 40.5% (*p* = 0.787) of patients with T1D with and without β-cell Tx reported weight gain and 40.0% vs. 42.5% (*p* = 0.399) reported less physical activity during the lockdown, with only 6.3% vs. 9.3% reporting weight loss and 4.4% vs. 10.0% increased physical activity (Figure 3; Supplementary Table S3). The changes in stress, anxiety, weight and physical activity in patients with β-cell Tx were not statistically significantly different between patients with different modalities of β-cell transplantation (Supplementary Table S4).

## Discussion

In this study, we show that islet or pancreas transplantation (β-cell Tx) in patients with type 1 diabetes leads to additional fear of infection, more stringent social isolation behavior and deterioration of glycemic control during the COVID-19 pandemic and the subsequent lockdown. In fact, having had a β-cell transplantation was the most important determinant of not leaving the house during the COVID-19 lockdown. In addition, patients with T1D both with and without β-cell Tx experience high rates of stress and anxiety, decreased physical activity and weight gain.

The COVID-19 pandemic is the most relevant recent example of a public health emergency of international concern. Patients with diabetes mellitus are considered a high-risk population during these situations. Indeed, diabetes mellitus is a key independent risk factor for a severe course of COVID-19. Patients with T1D have a high risk of developing severe COVID-19, with evidence pointing to an even higher risk as compared to patients with type 2 diabetes [6, 29–32]. Patients who are eligible for β-cell replacement therapy through either islet or whole pancreas transplantation usually have severely complicated T1D [19]. With glucose dysregulation and poor glycemic control significantly associated with severe COVID-19, this further increases their risk [7, 33]. In addition, although data on the specific risks in patients with β-cell Tx are currently scarce, patients with (solid) organ transplants have been marked as an essential risk group for a more severe course of COVID-19 as well. This higher risk for COVID-19 severity and mortality has been extensively linked to the use of immunosuppression [7, 8, 22, 34, 35]. Thus, patients who have received β-cell Tx for complicated T1D have multiple important factors adding up to an increasingly higher risk of severe COVID-19 and can therefore be considered a very vulnerable patient group.

These vulnerable patients at high risk for a severe course of COVID-19 were continuously warned to be particularly stringent in observing lockdown measures and practicing social isolation [9, 10]. Importantly, staying at home was strongly advised, but not mandatory during the lockdown in the Netherlands [25]. Nonetheless, almost half of the patients with β-cell Tx reported not leaving the house at all, which was almost three times higher than patients with T1D alone. This was associated with a significantly higher fear of COVID-19 infection in patients with β-cell Tx as compared to T1D. A history of CVD, as an additional risk factor for severe COVID-19, was also associated with not leaving the house. Other known risk factors for severe COVID-19, such as older age, male sex, a higher BMI, worse glycemic control, hypertension and pulmonary comorbidities were not found to be associated with isolating at home. Furthermore, we found high rates of stress and anxiety in this vulnerable patient group. This is in line with previous studies that have also described extensive psychological influences of the COVID-19 pandemic and lockdown measures in both patients with type 1 diabetes, and (solid) organ transplant recipients [4, 5, 10, 36–40].

Apart from the psychological impact, we also found a strong impact on physical outcomes with high rates of weight gain and decreased physical exercise during the COVID-19 lockdown. These unfavorable findings have been reported in the general population as well, and health effects appear to persist after lifting the lockdown [41–44]. Patients with β-cell Tx also showed a slight deterioration in glycemic control over the lockdown period. This could be related to the reported changes in daily structures, behavior, emotional distress, anxiety, weight gain and the limited possibility for physical exercise during the lockdown, as all of these factors are well-known to influence glycemic control [11–13, 37]. However, our analyses did not show these effects. Additionally, altered healthcare access due to a shift towards COVID-19-related care put greater emphasis on patients’ self-management [45], which may have complicated glycemic control. Patients with a higher pre-lockdown HbA1c were more likely to experience deterioration of HbA1c over the lockdown. Also, β-cell Tx recipients with a successful graft function were much more likely to experience deterioration of HbA1c as compared to patients with a failed graft. This may point to patients with a successful graft losing diabetes self-management skills as a result of their glycemic stabilization after transplantation, while patients with failed grafts continue to endure complicated diabetes [20].

Interestingly, in contrast to patients with β-cell Tx, patients with T1D showed a small overall improvement in glycemic control over the lockdown period [14]. This finding was supported by other smaller studies from Italy and Spain, which also found an improvement in glycemic control during lockdown in patients with T1D [15–18]. The difference in glycemic control between patients with T1D with and without β-cell Tx may be related to differences in self-management skills and social isolation behavior [24], the higher general impact of the pandemic and subsequent lockdown, and the increased (feeling of) vulnerability in patients with β-cell Tx, since in addition to having complicated T1D and using immunosuppression, these patients also more often have other (cardiovascular) comorbidities.

There are several strengths and limitations to our study. To our knowledge, this is the first study describing the psychological and physical impact of the COVID-19 pandemic and subsequent lockdown in patients with β-cell Tx, assessing a wide variety of outcomes including behavior, anxiety and stress, physical activity and weight. We determined the effect on glycemic control using both HbA1c as well as glucose monitoring data and included a large control group of patients with T1D without β-cell Tx. HbA1c measurements were conducted at median 9.3 weeks after the start of the lockdown. With HbA1c reflecting glycemic control over the preceding 8–12 weeks, interference of pre-lockdown glycemic control may be present [46]. Because of COVID-19 restrictions and altered healthcare access, we had to rely on patient-reported data for certain outcomes. This included weight change over the lockdown period. However, since reported weight change is often an underestimation of the actual weight change [47], the proportion of patients with weight gain may even be larger than reported in this current study. We used validated questionnaires for e.g., perceived stress, but could not compare them with pre-lockdown outcomes, because these questionnaires were not regularly used before the COVID-19 lockdown. For this reason, we asked patients in our additional questionnaire to compare outcomes like stress during the lockdown to before the lockdown. Using this approach, we were able to report changes in outcomes over the COVID-19 lockdown period. The COVID-19 pandemic has brought much attention to health inequalities, as certain groups were found to have been more highly impacted during the pandemic than others [48, 49]. In this study, we found no influence of sex or level of education, but we have no information on whether living in urban, suburban or rural communities or access to exercise possibilities may have played a role in our findings. We did not include a control group of people without risk factors for severe COVID-19, which could have provided us with information on a potential stepwise increasing impact of the lockdown with increasing vulnerability.

Lockdown measures are implemented to shield vulnerable population groups—including patients with β-cell Tx for T1D—from contracting COVID-19 in an attempt to prevent a severe course and mortality. However, during the lockdown this group experienced weight gain and deterioration of glycemic control. With both of these factors independently associated with a severe course of COVID-19 [6, 33], this pinpoints a complex problem wherein the effects of the lockdown may contribute to an even further increased risk of severe COVID-19. However, since poor glycemic control and increased weight are both modifiable risk factors, emphasis can be put on better (self-) management (support) as well as a healthy lifestyle.

This study was conducted during the first COVID-19 lockdown, when vaccines were not yet available. However, it is known that immunosuppressed transplant recipients often lack adequate antibody responses [50, 51]. A study in kidney transplant recipients showed similarly high rates of depression, anxiety and lower health-related quality of life after vaccination as compared to before vaccination, with only a small improvement in psychological distress [52]. These findings underline the continuing vulnerability of transplant recipients after vaccination, and may point to continued psychological, physical and behavioral impact.

In summary, patients with type 1 diabetes and a previous β-cell transplantation requiring immunosuppressive agents are at high risk during a public health emergency. Having multiple risk factors for a severe course of COVID-19, these patients and are highly impacted by the pandemic and subsequent lockdown. They experience high rates of fear, social isolation, worsening of glycemic control and weight gain which requires continuous awareness amongst healthcare professionals.

## Figures and Tables

**Table:** 

**Abbreviation**	Full term
**ACE**	Angiotensin converting enzyme
**ARB**	Angiotensin receptor blocker
**BMI**	Body mass index
**CGM**	Continuous glucose monitoring
**CI**	Confidence interval
**COPD**	Chronic obstructive pulmonary disease
**COVID-19**	Coronavirus disease 2019
**CV**	Coefficient of variation
**CVD**	Cardiovascular disease
**(e)GFR**	(estimated) Glomerular filtration rate
**FGM**	Flash glucose monitoring
**FSL**	FreeStyle Libre
**Hb**	Hemoglobin
**HbA1c**	Hemoglobin type A1c (glycosylated hemoglobin)
**IQR**	Interquartile range
**IS**	Immunosuppression
**ITx**	Islet transplantation
**IU**	International units
**IV**	Intravenously
**LDL**	Low-density lipoprotein
**LUMC**	Leiden University Medical Center
**MMF**	Mycophenolate mofetil
**mRNA**	Messenger ribonucleic acid
**NA**	Not applicable
**OR**	Odds ratio
**Pre-L**	Pre-lockdown
**Pred**	Prednisolone
**PSS**	Perceived Stress Scale
**PTx**	Pancreas transplantation
**Q1**	First quartile
**Q3**	Third quartile
**Ref.**	Reference
**SARS-CoV-2**	Severe acute respiratory syndrome coronavirus 2
**SD**	Standard deviation
**SPK**	Simultaneous pancreas-kidney transplantation
**T1D**	Type 1 diabetes mellitus
**Tac**	Tacrolimus
**TAR**	Time above range
**TBR**	Time below range
**TIR**	Time in range
**Tx**	Transplantation
**VAS**	Visual analogue scale

## Data Availability

The raw data supporting the conclusion of this article will be made available by the authors upon reasonable request, without undue reservation.
