# Supplementary material for: Impact of a Public Health Emergency on Behavior, Stress, Anxiety and Glycemic Control in Patients With Pancreas or Islet Transplantation for Type 1 Diabetes
Source: Transpl Int. 2024 Mar 27;37:12278. doi: 10.3389/ti.2024.12278 (PMC11005033; doi:10.3389/ti.2024.12278)
Supplement: Supplementary file 1 [file DataSheet1.docx]

**Supplementary Material**

**Supplementary figure S1 – Flowchart of study population**


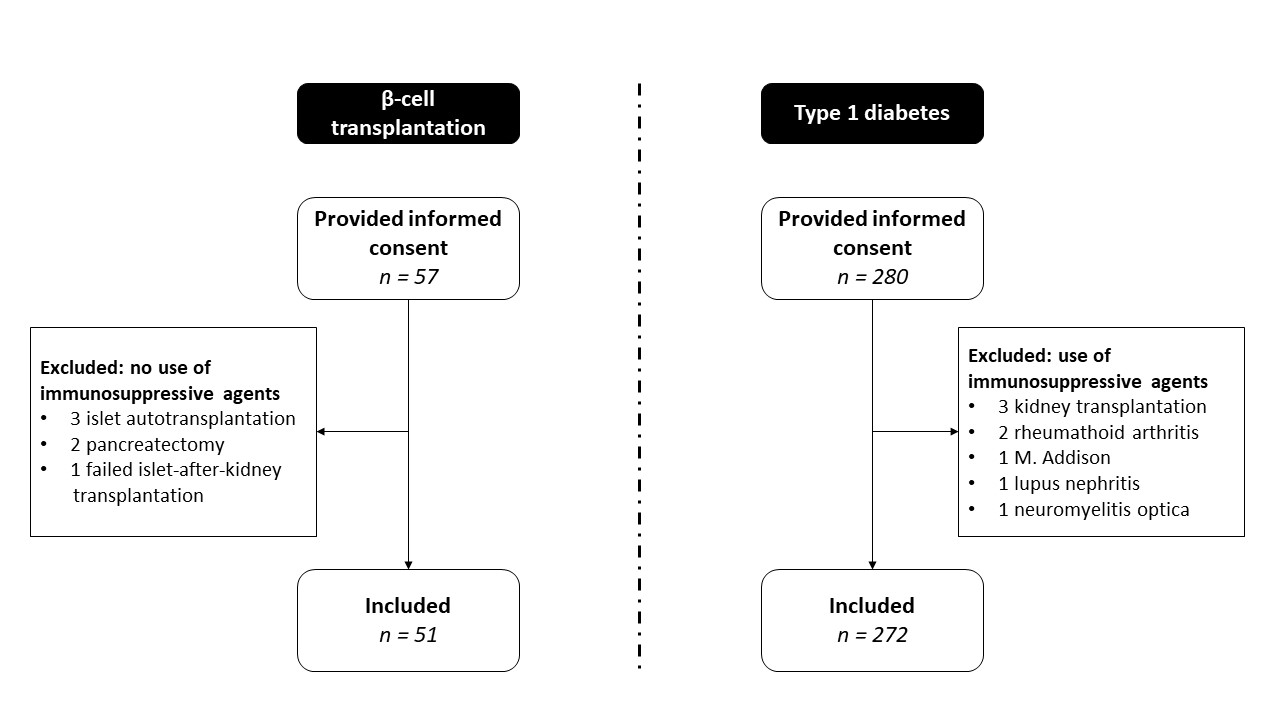


**Legend to Supplementary figure S1 – Flowchart of the study population**

Flowchart depicting the study population of pancreas and islet (β-cell) transplant recipients and the control population of patients with type 1 diabetes alone. All patients were originally recruited as part of a larger study, and signed written informed consent.^1^ For the current analysis, patients with a pancreas or islet a β-cell transplantation that did not use steroids and/or other immunosuppressive agents and patients with type 1 diabetes alone who did use steroids and/or other immunosuppressive agents were excluded. The reasons for exclusion are listed in the flowchart. In the subject with a failed islet-after-kidney transplantation, both the islet as well as the kidney graft had failed, for which reason immunosuppression was ceased.

**^1^** Ruissen MM, Regeer H, Landstra CP, et al. Increased stress, weight gain and less exercise in relation to glycemic control in people with type 1 and type 2 diabetes during the COVID-19 pandemic. *BMJ Open Diabetes Res Care.* 2021;9(1).

**Supplementary table S1 – Igls criteria for scoring β-cell graft function**

| **β-cell graft functional status** | **HbA1c**  % (mmol/mol) | **Severe hypoglycaemia**  events per year | **Insulin requirements**  U/kg/d | **C-peptide**  ng/mL (nmol/L) | **Treatment success** |
| --- | --- | --- | --- | --- | --- |
| Optimal | ≤ 6.5 (48) | None | None | > Baseline /  > 0.5 (0.17) | Yes |
| Good | < 7.0 (53) | None | < 50% baseline / < 0.5 | > Baseline / > 0.5 (0.17) | Yes |
| Marginal | ~ Baseline | < Baseline | ≥ 50% baseline /  ≥ 0.5 | > Baseline / > 0.5 (0.17) | No |
| Failure | ~ Baseline | ~ Baseline | ~ Baseline | ~ Baseline | No |
| **Adapted from:** Rickels MR, Stock PG, de Koning EJP, et al. Defining Outcomes for beta-cell Replacement Therapy in the Treatment of Diabetes: A Consensus Report on the Igls Criteria From the IPITA/EPITA Opinion Leaders Workshop. Transplantation. 2018;102(9):1479-1486.^29^ | | | | | |

**Supplementary methods S1 – Questionnaire on the impact of the COVID-19 lockdown on individual patients’ daily routines, glycaemic control, medication use, physical activity, weight, stress and anxiety (translated from Dutch)**

Since March 2020 the government has pronounced some rules and restrictions in order to halt the spreading of the coronavirus pandemic. From March 15^th^ on all Dutch citizens were asked to stay at home and work from home as much as possible and perform social distancing. We are interested in which way these rules and restrictions have impacted the lives of patients with diabetes, a high risk population according to the RIVM.

1. What is your highest completed or current education?

- Primary education (including special education)
- Practical training and education
- VMBO, LWOO, *V*SO (lower preparatory vocational education)
- MBO (senior secondary vocational education)
- HBO (higher professional education)
- University-level education, including (post-)doctoral studies
- Other, namely: ____________________

1. How do you test your blood glucose levels?

- Using fingerstick measurements
- Using a FreeStyle Libre or continuous glucose monitoring system
- I do not test my blood glucose levels

1. Do you feel like your glucose regulation has changed during the period of self-quarantine?

- No, my glucose regulation remained the same
- Yes (choose one of the options below)
- Keeping my glucose values stable is much easier
- Keeping my glucose values stable is somewhat easier
- Keeping my glucose values stable is somewhat more difficult
- Keeping my glucose values stable is a much more difficult

1. Did the amount of insulin you use change during the period of self-quarantine?
   *(Only applicable for patients using insulin to regulate their diabetes)*

- No, I use the same amount of insulin as before
- Yes (choose one of the options below)
  - I use much more insulin
  - I use somewhat more insulin
  - I use somewhat less insulin
  - I use much less insulin

1. Do you feel like your weight has changed during the period of self-quarantine?

- No, my weight remained the same
- Yes (chose on of the options below)
  - I gained weight
    - 1-2 kilograms
    - 3-4 kilograms
    - ≥ 5 kilograms
  - I lost weight
    - 1-2 kilograms
    - 3-4 kilograms
    - ≥ 5 kilograms

1. On a scale from 1-10, how anxious have you been to get infected with the coronavirus during the last 6 weeks?

VAS-scale 1-10

1. Have you experienced a change in stress since the start of the period of self-quarantine?

- No, my stress level remained the same
- Yes (chose one of the options below)
  - I experienced much less stress
  - I experienced somewhat less stress
  - I experienced somewhat more stress
  - I experienced much more stress

1. Have you experienced a change in anxiety since the start of the self-quarantine period?

- No, my anxiety level remained the same
- Yes (chose one of the options below)
  - I experienced much less anxiety
  - I experienced somewhat less anxiety
  - I experienced somewhat more anxiety
  - I experienced much more anxiety

1. How was your living situation prior to the period of self-quarantine? (chose one of the options below)

- I lived alone
- I lived with my partner
- I lived with my partner and children
- I lived with my children
- I lived with my parents
- I lived with my roommates

1. Did anything change regarding your exercise activities?

- No, my exercise activities remained the same
- Yes
- I exercised less than before
- I exercised more than before

1. On average, how many times did you leave your house/estate (including own garden) in the past 6 weeks?

- I do not go out of the house
- I only went out for work
- 1-2 times per day
- 3-5 times per day
- >5 times per day

1. On average, how many times did you visit a shop in the past 6 weeks?

- I do not go to shops anymore
- 1-2 times per week
- 3-5 times per week
- >5 times per week

1. On average, how many times did you receive visitors (including children) in the past 6 weeks?

- I did not receive any visitors
- 1-2 times per week
- 3-5 times per week
- >5 times per week

| **Supplementary table S2 – Impact of the COVID-19 lockdown on parameters of glycaemic control in patients with islet, solitary pancreas and simultaneous pancreas-kidney transplantation** | | | | |
| --- | --- | --- | --- | --- |
|  | **ITx**  *n = 14* | **PTx**  *n = 2* | **SPK**  *n = 17* | ***p*-value** |
| ΔHbA1c (mmol/mol Hb) | 2.3±6.7 | -9.5±3.5 | 2.5±10.0 | 0.180 |
|  | **ITx**  *n = 6* | **PTx**  *n = 0* | **SPK**  *n = 6* |  |
| ΔTAR (%) | 4.5 (-6.0 – 8.3) | NA | 5.5 (-2.8 – 9.3) | 0.872 |
| ΔTIR (%) | -4.0 (-5.3 – 3.5) | NA | -5.5 (-8.5 – 6.3) | 0.334 |
| ΔTBR (%) | 0.0 (-2.0 – 2.0) | NA | -0.5 (-2.5 – 0.0) | 0.270 |
| ITx = islet transplantation; PTx = solitary pancreas transplantation; SPK = simultaneous pancreas-kidney transplantation; TAR = time above range (% of time ≥ 10.0 mmol/L); TIR = time in range (% of time between 3.9 – 10.0 mmol/L; TBR = time below range (% of time < 3.9 mmol/L); NA = not available | | | | |

| **Supplementary table S3 – Impact of the COVID-19 lockdown on self-reported glycaemic control, insulin use, stress, anxiety, weight and physical exercise in patients with type 1 diabetes with and without β-cell transplantation** | | | | |
| --- | --- | --- | --- | --- |
|  | **All patients**  *n = 305* | **T1D**  *n = 257* | **β-cell Tx**  *n = 48* | ***p*-value** |
| Change in ability to regulate glucose (%) |  |  |  |  |
| *Easier* | 20.7% | 22.6% | 10.4% | **0.015** |
| *No change* | 48.9% | 46.7% | 60.4% |  |
| *More difficult* | 30.5% | 30.7% | 29.2% |  |
| Change in insulin use (%) |  |  |  |  |
| *Less insulin* | 8.1% | 8.2% | 7.3% | 0.871 |
| *No change in insulin* | 62.2% | 61.6% | 65.9% |  |
| *More insulin* | 29.7% | 30.2% | 26.8% |  |
| Change in stress (%) |  |  |  |  |
| *Less stress* | 22.0% | 21.8% | 22.9% | 0.984 |
| *No change in stress* | 44.6% | 44.7% | 43.8% |  |
| *More stress* | 33.4% | 33.5% | 33.3% |  |
| Change in anxiety (%) |  |  |  |  |
| *Less anxiety* | 19.0% | 18.3% | 22.9% | 0.677 |
| *No change in anxiety* | 53.1% | 54.1% | 47.9% |  |
| *More anxiety* | 27.9% | 27.6% | 29.2% |  |
| Change in weight (%) |  |  |  |  |
| *Weight loss* | 8.9% | 9.3% | 6.3% | 0.787 |
| *No change in weight* | 50.5% | 50.2% | 52.1% |  |
| *Weight gain* | 40.5% | 40.5% | 41.7% |  |
| Change in physical exercise (%) |  |  |  |  |
| *Less exercise* | 42.0% | 42.5% | 40.0% | 0.399 |
| *No change in exercise* | 48.9% | 47.5% | 55.6% |  |
| *More exercise* | 9.1% | 10.0% | 4.4% |  |
| T1D = type 1 diabetes; β-cell Tx = β-cell transplantation  *p-*value for differences between T1D and Tx | | | | |

| **Supplementary table S4 – Impact of the COVID-19 lockdown on self-reported glycaemic control, insulin use, stress, anxiety, weight and physical exercise in patients with islet, solitary pancreas and simultaneous pancreas-kidney transplantation** | | | | |
| --- | --- | --- | --- | --- |
|  | **ITx**  *n = 17* | **PTx**  *n = 4* | **SPK**  *n = 27* | ***p*-value** |
| Change in ability to regulate glucose (%) |  |  |  |  |
| *Easier* | 11.8% | 25.0% | 7.4% | 0.122 |
| *No change* | 58.8% | 0.0% | 70.4% |  |
| *More difficult* | 29.4% | 75.0% | 22.2% |  |
| Change in insulin use (%) |  |  |  |  |
| *Less insulin* | 5.9% | 25.0% | 5.0% | 0.417 |
| *No change in insulin* | 70.6% | 25.0% | 70.0% |  |
| *More insulin* | 23.5% | 50.0% | 25.0% |  |
| Change in stress (%) |  |  |  |  |
| *Less stress* | 23.5% | 25.0% | 22.2% | 0.380 |
| *No change in stress* | 47.1% | 0.0% | 48.1% |  |
| *More stress* | 29.4% | 75.0% | 29.6% |  |
| Change in anxiety (%) |  |  |  |  |
| *Less anxiety* | 23.5% | 25.0% | 22.2% | 0.232 |
| *No change in anxiety* | 47.1% | 0.0% | 55.6% |  |
| *More anxiety* | 29.4% | 75.0% | 22.2% |  |
| Change in weight (%) |  |  |  |  |
| *Weight loss* | 0.0% | 25.0% | 7.4% | 0.125 |
| *No change in weight* | 64.7% | 0.0% | 51.9% |  |
| *Weight gain* | 35.3% | 75.0% | 40.7% |  |
| Change in physical exercise (%) |  |  |  |  |
| *Less exercise* | 37.5% | 50.0% | 40.0% | 0.746 |
| *No change in exercise* | 62.5% | 50.0% | 52.0% |  |
| *More exercise* | 0.0% | 0.0% | 8.0% |  |
| ITx = islet transplantation; PTx = solitary pancreas transplantation; SPK = simultaneous pancreas-kidney transplantation | | | | |

| **Supplementary table S5 – Univariable and multivariable predictors of deterioration of HbA1c over the lockdown period in patients with β-cell transplantation** | | | | | | |
| --- | --- | --- | --- | --- | --- | --- |
|  | **Univariable logistic regression** | | | **Multivariable logistic regression** | | |
|  | **B** | **R^2^ (95% CI)** | ***p*-value** | **B** | **R^2^ (95% CI)** | ***p*-value** |
| Age, years | -0.009 | 0.991 (0.911 – 1.078) | 0.831 |  |  |  |
| Sex (ref. female) | 0.310 | 1.364 (0.356 – 5.217) | 0.651 |  |  |  |
| BMI, kg/m^2^ | -0.122 | 0.885 (0.743 – 1.055) | 0.885 |  |  |  |
| Level of education (ref. middle)  Low  High | 0.539  -0.161 | 1.714 (0.336 – 8.019)  0.852 (0.509 – 1.424) | 0.494  0.540 |  |  |  |
| HbA1c pre-L, mmol/mol Hb | -0.085 | 0.918 (0.858 – 0.983) | **0.014** | -0.078 | 0.925 (0.834 – 1.023) | 0.141 |
| Change in stress | 0.407 | 1.502 (0.613 – 3.681) | 0.374 |  |  |  |
| Change in anxiety | 0.013 | 1.013 (0.400 – 2.565) | 0.978 |  |  |  |
| Change in physical exercise | -0.223 | 0.800 (0.207 – 3.088) | 0.746 |  |  |  |
| Change in weight | -1.233 | 0.291 (0.075 – 1.138) | 0.076 |  |  |  |
| Igls score treatment success  (ref. failure) | 1.718 | 5.571 (1.297 – 23.934) | **0.021** | 0.216 | 1.241 (0.119 – 12.925) | 0.857 |
| VAS fear of infection | -0.225 | 0.799 (0.597 – 1.069) | 0.131 |  |  |  |
| Ref. = reference; BMI = body mass index; Hb = haemoglobin; pre-L = pre-lockdown; β-cell Tx = β-cell transplantation; T1D = type 1 diabetes mellitus; VAS = visual analogue scale | | | | | | |
